# High Hydrostatic Pressure‐Induced Changes in Carioca Bean Protein: Structural and Techno‐Functional Insights

**DOI:** 10.1111/1750-3841.70635

**Published:** 2025-11-02

**Authors:** Fabiana Helen Santos, Ludmilla de Carvalho Oliveira, Dirceu de Sousa Melo, Serafim Bakalis, Marcelo Cristianini

**Affiliations:** ^1^ Department of Food Engineering and Technology School of Food Engineering Universidade Estadual de Campinas Campinas São Paulo Brazil; ^2^ Department of Food Science University of Copenhagen Copenhagen Denmark; ^3^ Department of Biology Federal University of Lavras Lavras Minas Gerais Brazil

**Keywords:** emerging technologies, foaming properties, high‐pressure processing, plant‐based, surface hydrophobicity

## Abstract

Carioca bean protein concentrate (CBPC) has become a noteworthy alternative in the food industry. To expand their utilization in plant‐based food products, this study examined the effects of high hydrostatic pressure (HHP) treatment at 200–600 MPa for 10 min on the structural, techno‐functional, and color properties of CBPC. HHP treatment significantly influenced the structural and techno‐functional properties of CBPC. Particle size increased with pressure, reaching the largest volume‐weighted mean diameter (29.2 µm) at 400 MPa, accompanied by the lowest zeta potential (34.6 mV), indicative of protein aggregation. At 600 MPa, the pressure‐induced breakdown of aggregates resulted in a reduced particle size (14.7 µm) and enhanced exposure of hydrophobic groups. The surface hydrophobicity (*H*_0_) was significantly higher at 600 MPa, leading to increased enthalpy (3.4 J/g) and intrinsic fluorescence. Foaming properties were notably improved at 600 MPa, with an increase in foam capacity (35%) and foam stability (8%). Conversely, treatment at 200 MPa resulted in lower *H*_0_ values, associated with higher protein aggregation and reduced solubility (59%) compared to higher pressure treatments (64%–70%). HHP treatment, particularly at 600 MPa, effectively modifies the structural and functional properties of CBPC, enhancing its potential for use in aerated or foamed food products. Overall, almost all CBPC samples demonstrated emulsion stability close to 100%, surpassing widely used pulse proteins in the food industry. These findings support the use of HHP‐treated CBPC as a versatile ingredient in plant‐based food applications.

## 1 Introduction

The imminent challenge of feeding a global population projected to surpass 9 billion by 2050 highlights the critical need for increased food production and distribution (Yong et al. 2021). Currently, the global protein demand for 7.3 billion people is around 202 million tons, a figure expected to significantly escalate with population growth (Henchion et al. 2017). Beyond demographic shifts, rising concerns about the sustainability, health, and environmental impact of animal protein sources are propelling both the market and consumers to explore alternatives, such as plant‐based protein products (Bou et al. 2022; Y. Ma et al. 2023). In 2018, the global market for plant‐based protein products was valued at $35 billion, and the projection is that it will reach $45 billion by 2023 (Akharume et al. 2021).

At present, soy and wheat are the main protein sources used in plant‐based products. However, concerns about potential allergic reactions to these proteins have led many consumers to look for alternative sources (Avelar et al. 2021). As a result, some pulses are gaining prominence. Approximately 73 million metric tons of pulses are produced worldwide, with more than 70% of this production comprised of dry beans, lentils, chickpeas, and dry peas (Byanju and Lamsal 2021).

After soy, common beans (*Phaseolus vulgaris*) stand out as one of the most widely cultivated pulse crops, boasting a global production of approximately 28 million tons in 2021, with Brazil accounting for over 10% of this production (FAO 2023). Common beans, recognized as nonallergenic and nongenetically modified organisms, contain 20%–30% protein and serve as a substantial source of fiber, vitamins, minerals, and other bioactive compounds (Alfaro‐Diaz et al. 2021; Los et al. 2020). Among the main cultivars, carioca beans stand out for being the most produced in Brazil (Gouvêa et al. 2023), commanding 70% of production (de Oliveira et al. 2023). The processing of common beans into protein concentrates presents significant potential for the plant‐based segment and may reduce the dependency of national industries on imported soy and pea protein isolates and concentrates for alternative protein product development (Gouvêa et al. 2023).

Currently, numerous challenges persist in formulating alternative vegetable proteins sourced from pulses, particularly concerning techno‐functional aspects (Fernando 2022; Tang et al. 2021). Actions focused on improving the properties of the protein ingredients can significantly expand their application in plant‐based food products (Alfaro‐Diaz et al. 2021). In a recent study, we investigated structural modifications in carioca bean proteins induced by dynamic high pressure (DHP) (Santos et al. 2024), a technology that, unlike high hydrostatic pressure (HHP), is based on intense shear forces, turbulence, and cavitation phenomena that generate high pressure during the passage of the fluid through a narrow valve. In that study, changes in protein structure were observed, which consequently impacted their technological properties. These findings provided strong evidence that carioca bean proteins can be improved through innovative processing technologies. In line with these observations, the present study using HHP demonstrates another promising approach, reinforcing the potential of applying emerging high‐pressure technologies to enhance the functionality of this underutilized Brazilian protein source.

HHP can induce protein denaturation (especially in globular proteins), promoting the reorganization of inter‐ and intramolecular interactions and leading to new conformations that can result in modified techno‐functional properties, depending on the protein type, pressure magnitude, and processing duration (Hall and Moraru 2021). Importantly, these changes can occur without compromising food quality, sensory attributes, or nutritional properties (Alfaro‐Diaz et al. 2021).

Therefore, the aim of this study was to evaluate the impact of HHP, a technology not previously investigated on carioca bean protein concentrate (CBPC), on its structural characteristics in a pressure‐dependent manner, and subsequently, to analyze the resulting modifications in its techno‐functional and color properties. Such critical knowledge is essential both to advance the state of the art and to optimize the use of bean proteins tailored to specific functional requirements in food applications.

## 2 Materials and Methods

### 2.1 Material

The micronized carioca bean flour (CBF) used to produce the CBPC was supplied by the company R&S Blumos (Cotia, SP, Brazil). The flour composition on a dry basis was as follows: 44.61 g/100 g of carbohydrates, 44.06 g/100 g of protein, 6.75 g/100 g of ash, and 4.58 g/100 g of lipids. The moisture content was 8.26 g/100 g. The other reagents utilized during the analyses were of analytical grade.

### 2.2 Production of CBPC

The extraction of proteins from carioca beans was performed through the alkaline solubilization and isoelectric precipitation method, following the procedure proposed by Shevkani et al. (2015), with slight adjustments. CBF was suspended in distilled water at a 1:8 (w/v) flour‐to‐water ratio. The suspension's pH was adjusted to 9.0 using 1 M NaOH, followed by vigorous stirring for 1 h at 25°C. Subsequently, the mixture was centrifuged at 8000 × *g* for 20 min at 4°C, and the supernatant was separated. Protein precipitation was induced by adjusting the supernatant to pH 4.5 with 1 M HCl. The precipitated proteins were recovered by a second centrifugation under the same conditions (8000 × *g*, 20 min, 4°C). The obtained pellet was then resuspended in water and brought to neutral pH (7.0) using NaOH. Finally, the material was freeze‐dried (Liotop LP820, Liobras, Brazil) for approximately 4 days, and the resulting CBPC was stored at −18°C in sealed plastic bags until further analysis. The proximate composition of the CBPC on a dry basis is outlined as follows: 73.80 g/100 g of protein, 10.77 g/100 g of carbohydrates, 8.32 g/100 g of lipids, and 7.11 g/100 g of ash. The moisture content was 4.17 g/100 g (Santos et al. 2024).

### 2.3 HHP Treatment

A 5% (w/v) dispersion of CBPC was prepared in deionized water and stirred magnetically for 30 min. Then, the pH was corrected to 7.0 using NaOH (1 M). The dispersion was divided into portions and vacuum‐packed in polyethylene plastic bags. Samples were double‐sealed to prevent leakage. HHP treatment was carried out using a QFP 2L‐700 (Avure Technologies, OH, USA) with water as a transmitting medium. The samples were processed at 200, 300, 400, 500, and 600 MPa for 10 min. The pressure levels were reached within 1:10–02:30 min, and decompression was instantaneous. The temperature inside the pressure vessel during HHP processing ranged from an initial 27–30°C, reaching a maximum of 32°C, regulated through equipment temperature control. The HHP process was performed in duplicate. The treated samples were freeze‐dried (Liotop LP820, Liobras, Brazil) and then stored at −18°C until analysis. An untreated sample (control) was also prepared at pH 7.0 under environmental conditions. The samples were investigated as follows.

### 2.4 Structural Properties

The structural analyses included particle size distribution (PSD), zeta potential, differential scanning calorimetry (DSC), and Fourier transform infrared spectroscopy (FTIR). PSD and zeta potential were assessed for the control and all treatment conditions (200, 300, 400, 500, and 600 MPa), whereas DCS and FTIR analyses were performed only for the control and the CBPC treated at 200, 400, and 600 MPa. This streamlined approach effectively reduced the overall number of experiments, simplifying the study's scope without compromising the understanding of significant structural changes resulting from the treatments.

#### 2.4.1 Particle Size Distribution

The PSDs of CBPC samples (HHP‐treated CBPC and control) were performed at room temperature (25°C) by a static laser diffraction analyzer (Mastersizer 3000 Hydro, Malvern Instruments, UK). Before measurement, samples were diluted in deionized water at a 1:100 (w/v) ratio, stirred for 30 min, and their pHs were adjusted to 7.0. Each protein concentrate was evaluated in duplicate, with six measurements obtained per replicate. The parameters determined included the surface mean diameter [*D*(3,2)], volume mean diameter [*D*(4,3)], and the percentiles *D*10, *D*50, and *D*90. The refractive indices of 1.45 (particles) and 1.33 (dispersing medium) were applied.

#### 2.4.2 Surface Charge (Zeta Potential)

The surface charge of the samples was evaluated at 20°C utilizing a Zetasizer nano‐ZS (Malvern Instruments Ltd., UK). CBPC dispersions previously prepared for particle size measurements were further diluted in Milli‐Q water to a final concentration of 0.1% (w/v). Zeta potential was assessed in two independent replicates, each measured in triplicate.

#### 2.4.3 Differential Scanning Calorimetry

Thermal properties of control and HHP‐treated CBPC samples, including denaturation temperature peak (*T*_d_, °C), enthalpy of transition (Δ*H*), and onset/endset temperatures (*T*_onset_ and *T*_endset_, °C), were analyzed by a differential scanning calorimeter (DSC1, STARe System, Mettler Toledo, UK). Around 9 mg of each sample was placed in an aluminum crucible, which was hermetically sealed and subjected to heating from 20 to 120°C at a constant rate of 5°C/min. An empty and sealed crucible served as the reference. All measurements were carried out in duplicate.

#### 2.4.4 Fourier Transform Infrared Spectroscopy

FTIR spectra for the control and treated CBPC were obtained by the Fourier Transform spectrometer (MB100, ABB Bomem, Canada), operating in the range of 500–3500 cm^−1^, with 64 scans and a spectral resolution of 4 cm^−1^.

#### 2.4.5 Preparation of Protein Stock Solutions

Supernatants used for surface hydrophobicity and intrinsic fluorescence analyses were prepared from CBPC dispersions (3 mg protein/mL) of control and high‐pressure‐treated samples (200, 400, and 600 MPa), following the method described by Santos et al. (2024). The resulting supernatants’ protein concentration was quantified using a NanoDrop 1000 spectrophotometer (Thermo Fisher Scientific, USA).

##### 2.4.5.1 Surface Hydrophobicity (*H*_0_)

The surface hydrophobicity (*H*_0_) of CBPC samples was evaluated following the methodology detailed by Los et al. (2020) with minor adjustments. Initially, protein solutions (0.002–0.01 mg/mL) were prepared by diluting stocks in phosphate buffer (10 mM, pH 7.0). To each 1 mL protein solution, 5 µL of an 8 mM ANS solution (in 10 mM phosphate buffer) was added and vortexed for 10 s. Fluorescence intensity (FI) was measured at 20°C by a microplate spectrofluorometer (SpectraMax i3, Molecular Devices, USA) with excitation at 390 nm, emission at 470 nm, and 5 nm slit widths. FI values were corrected by subtracting the signal from protein samples without ANS. The slope of the FI versus protein concentration curve, obtained by linear regression, was used to estimate *H*_0_. All measurements were performed at least twice.

##### 2.4.5.2 Intrinsic Fluorescence

Intrinsic fluorescence was evaluated with a SpectraMax i3 microplate reader (Molecular Devices, USA), using protein solutions diluted to 0.02 mg/mL in phosphate buffer. Tyrosine and tryptophan residues were excited at 275 nm, and emission was monitored from 295 to 500 nm at 5 nm intervals. Analyses were done in triplicate for both control and HHP‐treated samples.

### 2.5 Techno‐Functional Properties

#### 2.5.1 Protein Solubility

Protein solubility was assessed following the method outlined by Calderón‐Chiu et al. (2021) with minor alterations. A 25 mg CBPC sample was mixed with 25 mL of distilled water, stirred for 30 min, and pH‐adjusted to 7.0 using 1 M NaOH. The dispersion was centrifuged (7500 × *g*, 15 min), and the supernatant protein content was quantified by the Bradford assay (Bradford 1976) with bovine serum albumin (BSA) as a standard. The Dumas method (*N* × 6.25) was used to determine total protein, and solubility (%) was calculated using the following equation:

(1) $$\mathrm{Solubility}\;\left(\%\right)=\frac{\mathrm{Protein}\;\mathrm{content}\;\mathrm{in}\;\mathrm{supernatant}}{\mathrm{Total}\;\mathrm{protein}\;\mathrm{content}\;\mathrm{in}\;\mathrm{the}\;\mathrm{sample}}\times 100$$

#### 2.5.2 Water Holding Capacity (WHC) and Oil Holding Capacity (OHC)

Water holding capacity (WHC) and oil holding capacity (OHC) were evaluated on the basis of the methods proposed by Rodríguez‐Ambriz et al. (2005) and Lin and Zayas (1987), respectively, both with adaptations as described by Ogunwolu et al. (2009). A 0.1 g CBPC sample was vortexed with 1 mL of distilled water or oil for 30 s. For WHC, the sample was centrifuged (1800 × *g*, 20 min, 25°C), and the supernatant was drained at a 45° angle for 10 min. For OHC, the mixture stood for 30 min at room temperature, followed by centrifugation (13,600 × *g*, 10 min, 25°C) and draining under the same conditions. WHC and OHC were calculated as follows:

(2) $$\mathrm{WHC}\;\left(g_{\mathrm{water}}/g_{\mathrm{CBPC}}\right)=\frac{W_{2}-W_{1}}{W_{0}}$$

(3) $$\mathrm{OHC}\;\left(g_{\mathrm{oil}}/g_{\mathrm{CBPC}}\right)=\frac{W_{2}-W_{1}}{W_{0}}$$

where *W*_2_ represents the weight of the microtube and sample post‐absorbing water or oil; *W*_1_ refers to the initial weight of the microtube and sample; and *W*_0_ corresponds to the sample's initial dry weight.

#### 2.5.3 Emulsifying Properties

The emulsifying properties were evaluated by the spectrophotometric method proposed by Pearce and Kinsella (1978), emulsifying activity index (EAI), and by analysis of emulsion capacity (EC) and stability (ES) described by Karaman et al. (2022).

To assess the EAI, 0.3 g of the CBPC sample was mixed in 30 mL of distilled water, with the pH adjusted to 7.0. Subsequently, 10 mL of sunflower oil was incorporated, and the mixture was emulsified (20,000 rpm, 1 min) by an Ultra‐Turrax homogenizer (T25‐digital, IKA, Germany). Immediately after emulsification (*T*_0_), a 50 µL sample was drawn from the bottom and transferred into 10 mL of 0.1% SDS solution. Absorbance was then measured at 500 nm with a spectrophotometer (Beckman Coulter DU 800, USA), and the EAI was calculated according to the following equation:

(4) $$\mathrm{EAI}\;\left(m^{2}/g\right)=\frac{2\;\times \;2303\;\times \;A_{0}\times \;N}{10,000\;\times \;\theta \;\times \;L\;\times \;C}$$

where *A*_0_ represents the absorbance at 500 nm, *N* is the dilution factor (200), θ corresponds to the oil phase fraction (0.25), *L* is cuvette thickness (0.01 m), and *C* is the protein concentration in the dispersion (g/mL).

For EC evaluation, one dispersion with 0.25 g of CBPC and 5 mL of distilled water was prepared in Falcon tubes, with pH adjustment to 7.0 using NaOH (1 M). Subsequently, 5 mL of sunflower oil was added, and the mixture was homogenized at 10,000 rpm for 1 min. Emulsions were centrifuged for 15 min at 3000*g*, and the height of the emulsion layer (mL) and the total tube volume (mL) were recorded. The EC was calculated by the following equation:

(5) $$\mathrm{EC}\;\left(\%\right)=\left(\frac{V_{1}}{V_{3}}\right)\times 100$$

The tubes were then incubated at 80°C for 30 min, followed by sequential cooling steps: first in an ice bath and then under running water. Afterwards, the tubes were centrifuged once again at 3000*g* for 15 min. ES was determined using the following equation:

(6) $$\mathrm{ES}\;\left(\%\right)=\left(\frac{V_{2}}{V_{3}}\right)\times 100$$

where $V_{1}$ is emulsion layer (mL);$V_{2}$ is the emulsion layer post‐incubation (mL); and $V_{3}$ is the total tube volume (mL).

#### 2.5.4 Foaming Capacity (FC) and Stability (FS)

FC and FS were assessed following the method outlined by Shevkani et al. (2015). CBPC samples were dispersed in distilled water (1% w/v, 20 mL, pH 7.0) within a graduated cylinder (50 mL). Subsequently, the dispersions were homogenized using an Ultra‐Turrax (20,000 rpm; 1 min). FC was determined on the basis of the percent increase in the suspension volume after mixing (Equation 7), whereas FS was assessed by measuring the percentage of foam retained after 30 min:

(7) $$\mathrm{FC}\;\left(\%\right)=\frac{V_{2}-V_{1}}{V_{1}}\times 100$$

(8) $$\mathrm{FS}\;\left(\%\right)=\frac{V_{t}}{V_{2}}\times 100$$

where $V_{1}$ refers to the initial dispersion volume, $V_{2}$ corresponds to the dispersion's volume post‐homogenization, and $V_{t}$ is the foam volume remaining after 30 min.

### 2.6 Color Properties

Color measurements of powdered CBPC samples were performed with a HunterLab UltraScan PRO colorimeter (Hunter Associate Laboratory Inc., USA) operating in reflectance mode. Analyses were carried out at room temperature using illuminant *D*65 and a 10° standard observer, with quadruplicate readings. The *L* ^*^, *a* ^*^, and *b* ^*^ values were obtained, where *L* ^*^ reflects lightness (0 = black to 100 = white), *a* ^*^ spans from green (−*a* ^*^) to red (+*a* ^*^), and *b* ^*^ from blue (−*b* ^*^) to yellow (+*b* ^*^). Additionally, chroma (*C* ^*^) and hue angle (*h*°) were calculated using the following equations, respectively:

(9) $$C^{*}=\sqrt{\left(a^{*2}+b^{*2}\right)}$$

(10) $$h^{\circ }=\left(b^{*}/a^{*}\right)$$

### 2.7 Statistical Analysis

The results were statistically assessed by analysis of variance (ANOVA), and treatment means were compared by Tukey's test at a 5% significance level via STATISTICA software (version 14.0). Data were expressed as means values ± standard deviation.

## 3 Results and Discussion

### 3.1 Structural Properties

#### 3.1.1 Particle Size Distribution

**FIGURE 1:**
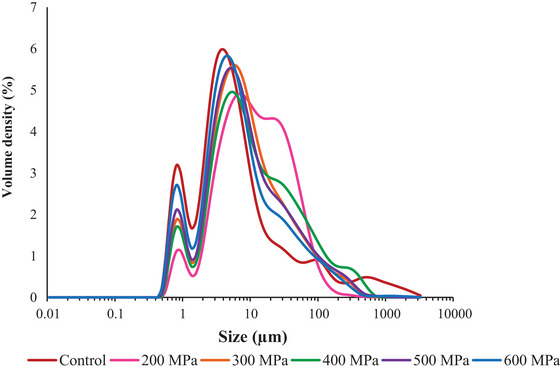
Particle size of control and HHP‐treated carioca bean protein concentrate (CBPC) at 200, 300, 400, 500, and 600 MPa.

**FIGURE 2:**
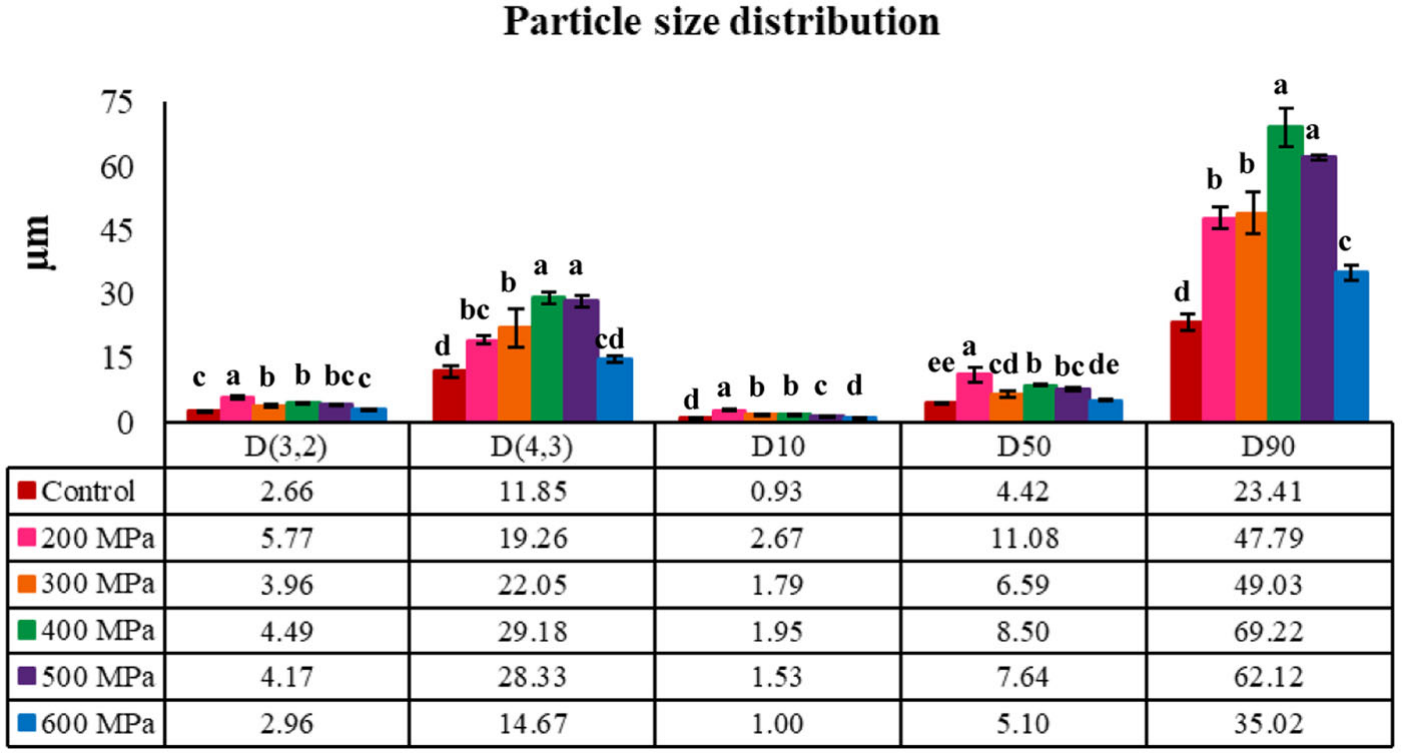
Effect of the high hydrostatic pressure on the particle size distribution of control and treated‐carioca bean protein concentrate (CBPC) at 200, 300, 400, 500, and 600 MPa. *D*(3,2): surface mean diameter; *D*(4,3): volume mean diameter; *D*10, *D*50, *D*90: cumulative diameter percentiles. Different letters denote statistically significant differences among samples treated at different high‐pressure levels (*p* < 0.05). Error bars represent standard deviation (SD).

The PSD of both the control and HHP‐treated samples is represented in Figures 1 and 2. The PSD of proteins can provide insights into their structure and aggregation, which influences their techno‐functional properties (Bing et al. 2023). Almost all the CBPC samples displayed a bimodal distribution, characterized by two distinct peaks: one at 1 µm and another around 4–6 µm (Figure 1). However, it is worth noting that the peaks of the samples treated at 200 and 400 MPa, particularly the second peak, were relatively lower compared to the other samples. In addition, the treatment at 200 MPa did not exhibit a well‐defined second peak, deviating slightly from the observed pattern. The second peak of the CBPC treated at 200 MPa became wider compared to the control and the other HHP‐treated CBPCs processed at higher pressures, suggesting a higher polydispersity of the particles and a greater presence of larger aggregates (Martínez et al. 2011).

The HHP up to 500 MPa resulted in a marked and cumulative increase in volume‐weighted mean diameter; however, beyond this pressure, a decreasing trend was observed (Figure 2). According to Perreault et al. (2017), HHP induces disruption and structural disorder of protein weak bonds, which promotes protein denaturation and aggregation. Under lower pressures (200–400 MPa), an aggregation of previously existing aggregates may have occurred, and then the previously formed aggregates were disrupted as pressure treatment increased to 600 MPa. At 600 MPa, the sample did not show a significant difference (*p* > 0.05) from the control. The same behavior was observed for the surface‐weighted mean diameter. Initially, the application of pressure led to an increase in the *D*(3,2) value. However, at 600 MPa, the value became similar to that of the control sample.

Regarding the percentile *D*50, it is interesting to note that 50% of the particles in the CBPC treated at 200 MPa had a PSD smaller than 11.10 µm, whereas the control and CBPC treated at 600 MPa had values lower than half (5.10 µm). In the study performed by Gouvêa et al. (2023), non‐processed common bean protein concentrate exhibited a similar *D*50 (5.23 µm). The higher *D*50 for the sample treated at 200 MPa is supported by the rightward shift of its second peak compared to the other samples (Figure 1). Conversely, larger particle diameters (*D*90) were not observed for CBPC treated at 200 MPa but for CBPC treated at 400 and 500 MPa. The greater polydispersity of the particles in the sample treated at 200 MPa, compared to the samples treated at 400 and 500 MPa, justifies its lower *D*90. Despite this observation, all concentrates can still be classified as a fine powder, because 90% of the particles (*D*90) are smaller than 70 µm. Besides that, these values are lower compared to unprocessed soy protein concentrate and isolate, as well as pea protein isolate (Gouvêa et al. 2023).

#### 3.1.2 Surface Charge (Zeta Potential)

**FIGURE 3:**
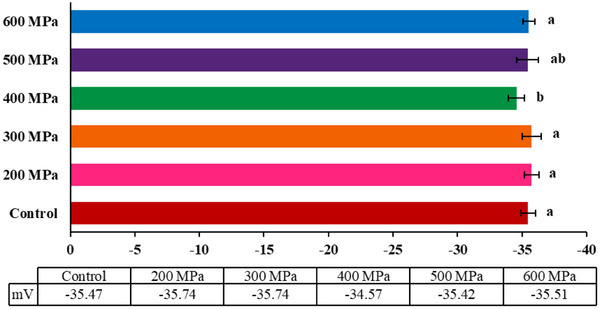
Zeta potential of control and treated‐carioca bean protein concentrate (CBPC) at 200, 300, 400, 500, and 600 MPa. Different letters denote statistically significant differences among samples treated at different high‐pressure levels (*p* < 0.05). Error bars represent standard deviation (SD).

The protein solutions’ zeta potential (ζ‐potential) is shown in Figure 3. ζ‐Potential reflects the surface charge of particles, playing a key role in their dispersion stability and aggregation behavior (Y. Zhao et al. 2023). The analysis was conducted at a neutral pH of 7.0, where negative electrostatic repulsive forces generally dominate (Sharan et al. 2022). The prevalence of negative charges accounts for the observed negative ζ‐potential in all the analyzed samples. Significant changes in surface charge (*p* < 0.05) were detected only at 400 MPa. At this pressure, the ζ‐potential decreased compared to the control sample and the other treated CBPC, except for the CBPC treated at 500 MPa. The decrease in ζ‐potential may be associated with denaturation and subsequent aggregation of proteins (Saricaoglu et al. 2018), which justifies the higher particle size of the sample treated at 400 MPa (Figure 2).

Commonly, a numerically low ζ‐potential indicates a reduced capacity of the protein to resist aggregation (Ong et al. 2022; Y. Ma et al. 2023). However, in this study, all protein solutions exhibited zeta potential superior to ±30 mV (Saricaoglu et al. 2017). Values of ζ‐potential above ±30 mV generally provide enough electrostatic repulsion to prevent particle aggregation and preserve the physical stability (Y. Ma et al. 2023). Furthermore, the absolute values found by CBPC samples were higher than pea, lentil, and other varieties of beans, indicating higher stability of this alternative protein source (Karaca et al. 2011; Ge et al. 2021).

The surface charge may influence protein functionality, especially emulsifying properties (Miranda et al. 2022). A greater absolute value of zeta potential may, for example, enhance the stability of emulsion by promoting stronger electrostatic repulsion between emulsion droplets (Burger and Zhang 2019).

#### 3.1.3 Differential Scanning Calorimetry

To investigate the impact of HHP application on sample temperatures (denaturation, onset, and endset) and enthalpy, Table 1 provides a concise summary of these parameters. A significant increase (*p* < 0.05) in denaturation temperature (*T*_d_) was observed in the CBPC samples treated by HHP when compared to the control. A greater *T*_d_ value is associated with a compact structure protein and a reduced hydrophilic property (Mulla et al. 2022). The increase of the *T*_d_ by the pressure application suggests the unfolding and aggregation of protein (Qin et al. 2013). According to Karaman et al. (2022), aggregated vicilin (main bean storage protein) would have higher *T*_d_ than the unaggregated. Similar behavior was observed for the *T*_onset_ and *T*_endset_, which means a rise in these temperatures after HHP treatment. The thermal stability (*T*_d_) of walnut protein isolate treated with high pressure also exhibited a significant increase (Qin et al. 2013).

**TABLE 1:** Thermal properties of the carioca bean protein concentrate (CBPC) untreated and treated at 200, 400, and 600 MPa.

Properties	Control	200 MPa	400 MPa	600 MPa
*T*_d_ (°C)	57.73 ± 0.93^b^	63.54 ± 0.51^a^	64.45 ± 0.38^a^	62.13 ± 0.01^a^
*T*_onset_ (°C)	47.56 ± 0.94^b^	50.64 ± 0.04^a^	50.67 ± 0.36^a^	50.09 ± 0.54^ab^
*T*_endset_ (°C)	64.42 ± 0.87^b^	71.34 ± 1.14^a^	72.55 ± 0.10^a^	68.97 ± 0.31^a^
Δ*H* (J/g^*^)	2.78 ± 0.15^b^	3.05 ± 0.02^ab^	3.26 ± 0.01^ab^	3.42 ± 0.13^a^

*Note*: Different superscript letters in the same line mean statistically significant differences between samples (*p* < 0.05). *T*_d_: Denaturation temperature.

* A similar moisture content was assumed across the samples.

Regarding the Δ*H*, almost all HHP‐treated CBPC samples exhibited values statistically similar to the control (*p* > 0.05), except the concentrate treated at 600 MPa. The Δ*H* reproduces the energy required to induce protein denaturation (Gouvêa et al. 2023). HHP can trigger protein unfolding, resulting in a more extended and disordered structure. This promotes protein destabilization and reduces the material's enthalpy (Dehnad et al. 2024). However, as observed in previous analyses, the application of 600 MPa pressure in this study likely disrupted previously formed protein aggregates and subsequently induced partial folding of CBPC. It is believed that folding is governed by stronger hydrophobic interactions within the sample, which suggests that more energy (as indicated by the highest enthalpy) is required to denature the CBPC treated at 600 MPa compared to the control sample (Gundogan and Karaca 2020). The highest Δ*H* indicates a greater thermal stability of this treated protein concentrate in comparison to the control.

#### 3.1.4 Fourier Transform Infrared Spectroscopy

**FIGURE 4:**
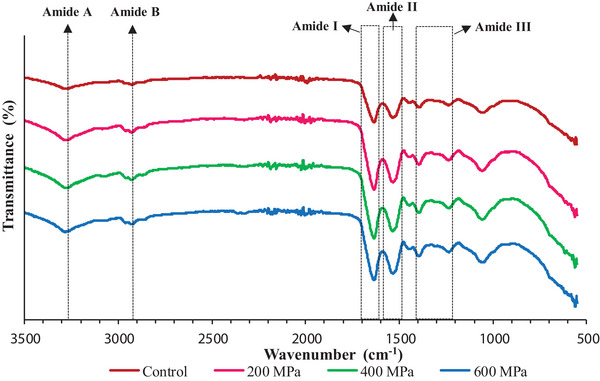
FTIR spectra in the 3500–500 cm^−1^ range for control and treated‐carioca bean protein concentrate (CBPC) at 200, 400, and 600 MPa.

As can be observed in Figure 4, the FTIR spectra of all CBPC presented a high degree of similarity, showcasing two prominent absorption bands: Amide I and Amide II (X. Zhao et al. 2023; Barth 2007). The Amide I region (1600–1700 cm^−1^) provides valuable information on the secondary structure of proteins in infrared spectroscopy, largely due to C═O stretching and H─O─H bending vibrations (Y. Zhao et al. 2023). According to the structural interpretation of the Amide I band, the CBPC is mainly made up of β‐sheet structures (1635 cm^−1^) (Dehnad et al. 2024; Tang et al. 2021; Yang et al. 2018). β‐Sheet regions are less responsive to pressure and more resistant to structural deformation than other structures, such as α‐helices (Queirós et al. 2018). This explains why the application of HHP did not cause any peak shifting in this region. Furthermore, the Amide II region (1480–1585 cm^−1^), which is associated with N─H bending and C─N stretching vibration, was identified in the CBPC by a peak around 1535 cm^−1^ (Gundogan and Karaca 2020).

Besides peaks at 1635 and 1535 cm^−1^, the control spectra also showed main absorption bands at approximately 3276, 2927, 1448, 1394, 1236, and 1057 cm^−1^. Careful observation revealed that HHP treatment in some cases slightly shifted the peaks at 3276, 2927, and 1394 cm^−1^, suggesting a few changes in terms of structure and conformation. At 200 and 400 MPa, the absorption peak in the control sample at 3276 cm^−1^ (amide A region) shifted to 3280 cm^−1^, whereas at 600 MPa, the shift was even greater, reaching 3285 cm^−1^. This region is produced by the O─H stretching and N─H bending vibrations and, according to Bing et al. (2023), can be associated with hydrogen bonds distributed along the polypeptide chain. The shift toward higher wavelengths suggests stronger bonds and perhaps conformational modifications of the molecules, such as aggregation, caused by interactions or hydrogen bonds between the proteins. Another slight shift was noted at the peak around 2927 cm^−1^ (amide B), which moved to 2925 cm^−1^ after high‐pressure treatments at 400 and 600 MPa. This wavelength is assigned to the asymmetric stretching vibration of C─H groups arising from carbohydrates, proteins, and neutral lipids (Gundogan and Karaca 2020). Moreover, the peak initially observed at 1394 cm^−1^ also shifted after the CBPC sample was subjected to 400 and 600 MPa (1396 cm^−1^). Defined as the Amide III region (1200–1400 cm^−1^), the observed peaks are indicative of interactions between proteins and macromolecules like carbohydrates (Gundogan and Karaca 2020). This finding aligns with the composition of CBPC, where carbohydrates and lipids are the other main constituents of the concentrates, accounting for 10.8 g/100 g and 8.32 g/100 g, respectively.

Overall, the application of HHP resulted in only subtle spectral changes, specifically in the amide A, B, and III bands, where slight peak shifts suggest conformational modifications and potential interactions between proteins and other macromolecules present in the concentrate. Moreover, the detected bands fall within the range described for other legumes in the literature (Ahmed et al. 2018), and the obtained spectra are consistent with those reported for distinct bean proteins (Gundogan and Karaca 2020; E. S. Tan et al. 2014).

#### 3.1.5 Surface Hydrophobicity (*H*_0_)

**FIGURE 5:**
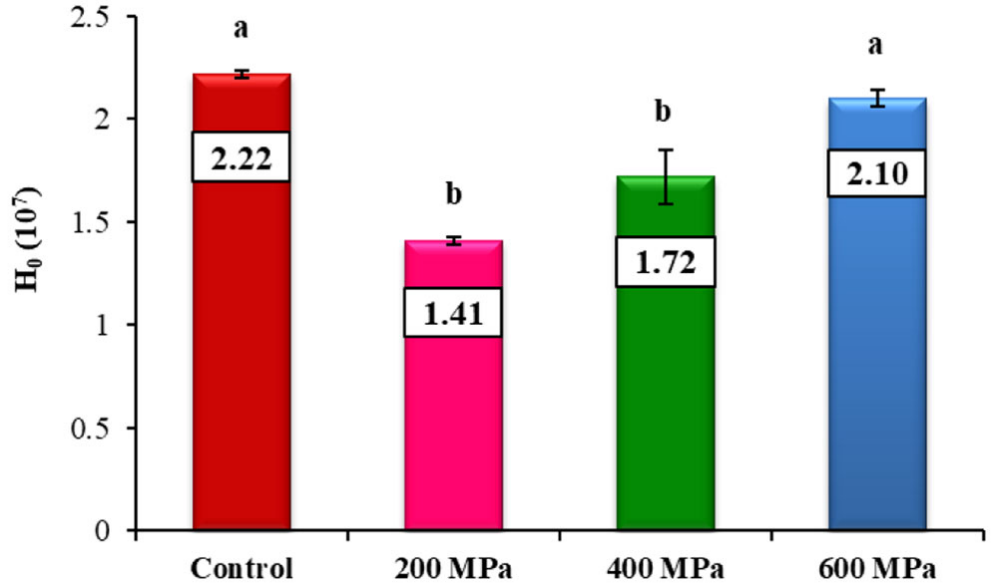
Surface hydrophobicity of untreated and treated carioca bean protein concentrate (CBPC) at 200, 400, and 600 MPa. Different letters denote statistically significant differences among samples treated at different high‐pressure levels (*p* < 0.05). Error bars represent standard deviation (SD).

As observed in other analyses, the treatments at lower pressures (200 and 400 MPa) resulted in a significant reduction (*p* < 0.05) in the surface hydrophobicity (*H*_0_) of CBPC samples (Figure 5) when compared to the control. However, at 600 MPa, the *H*_0_ value did not differ significantly from the control (*p* > 0.05). Pressure mainly affects proteins by disrupting non‐covalent interactions within and between protein molecules, followed by rearranging intra‐ and intermolecular bonds (Li and Wang 2019). The treatment at 200 and 400 MPa may have induced protein unfolding, resulting in greater exposure of hydrophobic groups typically hidden within the protein structure. As a result, there was an enhancement in the accessibility of hydrophobic groups, facilitating the fast formation of new interactions between protein molecules. These interactions, in turn, promoted the subsequent formation of aggregates (Queirós et al. 2018). According to Luo et al. (2022), the aggregation caused by HHP is reflected in a decrease in *H*_0_, because there is a reduced exposure of hydrophobic groups on the protein surface. On the other hand, applying higher pressure (600 MPa) may have triggered the breakdown of aggregates that were previously formed under lower pressure conditions, increasing the availability of hydrophobic groups on the protein surface and, consequently, the *H*_0_ value.

The opposite effect was reported by several other authors, such as Mulla et al. (2022) and Queirós et al. (2018). According to the authors, pressures up to 400 MPa generally increase the *H*_0_ of plant proteins; however, further treatment showed a decline in the hydrophobicity. These observations underscore that the conformational changes in proteins are not only influenced by the applied pressure but are also profoundly impacted by the specific protein source (Li and Wang 2019). Another critical factor in interpreting pressure effects on *H*_0_ is protein concentration, which affects the tendency of proteins to aggregate. At higher concentrations, as that used here (5%), aggregation is favored, decreasing the exposure of hydrophobic groups (Queirós et al. 2018; X.‐S. Wang et al. 2008).

#### 3.1.6 Intrinsic Fluorescence

**FIGURE 6:**
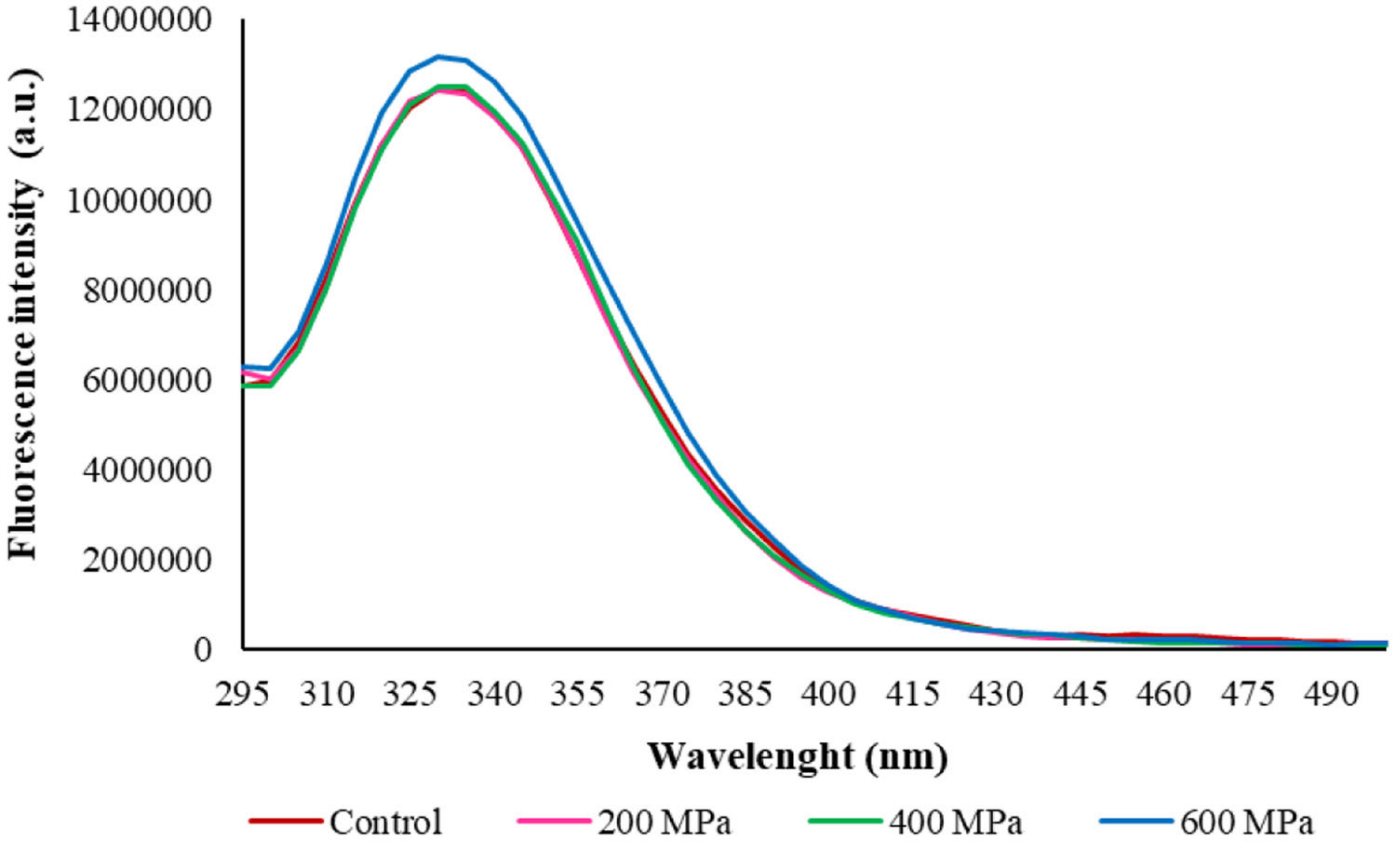
Intrinsic fluorescence of control and HHP‐treated carioca bean protein concentrate (CBPC) at 200, 400, and 600 MPa.

Intrinsic fluorescence is a useful method for monitoring changes in protein structure. Evaluating changes in the shape, position, and intensity of the emission spectra can provide valuable insights into protein unfolding and oligomerization (Mune et al. 2020). As can be seen in Figure 6, all the CBPC samples exhibited similar shapes and maximum emissions wavelengths (*λ*_max_), 330 nm. *λ*_max_ around 330 nm is characteristic of tryptophan residues situated in hydrophobic environments, like the interior of globulin proteins (Yin et al. 2008; K. Wang et al. 2017). Another possibility is the unavailability of the residues due to the aggregate's formation. Conversely, the FI of CBPC treated at 600 MPa was slightly higher than the other CBPC samples. Higher FI suggested a greater exposure of Tyr and Trp residues on the protein surface. The rise in FI after HHP processing may be linked to several factors, such as alterations in the microenvironment, exposure to hydrophobic residues, and protein aggregation (Dehnad et al. 2024). In this study, the exposition of the hydrophobic residues at 600 MPa is likely associated with the disruption of larger aggregates that were previously formed at 200 and 400 MPa.

### 3.2 Techno‐Functional Properties

#### 3.2.1 Protein Solubility

Except for the CBPC treated at 200 MPa, the application of HHP did not have a significant impact (*p* > 0.05) on the solubility of the CBPC samples (Table 2). At 200 MPa, the solubility of CBPC was significantly lower, measuring less than 59%. This decrease can be attributed to the increase in hydrophobic interactions, which promote interactions between proteins, thereby reducing solubility (Damodaran 2008). Indeed, the treatment at 200 MPa triggered the lowest *H*_0_ among the protein concentrates (Figure 5). Furthermore, a higher number of aggregates were possibly formed at this pressure (Figure 1), which may explain its lower solubility. A higher number of aggregates reduces the capacity of water to penetrate the protein's interior and bind to polar residues, reducing the solubility. Likewise, Lin and Fernández‐Fraguas (2020) identified a significant reduction in the solubility of pinto bean protein concentrate processed at 450 MPa for 10 min, reinforcing that HHP can compromise the aqueous solubility of bean proteins, depending on the pressure level and treatment time.

**TABLE 2:** Techno‐functional properties of carioca bean protein concentrate (CBPC) untreated and treated at 200, 300, 400, 500, and 600 MPa.

Properties	Control	200 MPa	300 MPa	400 MPa	500 MPa	600 MPa
Solubility (%)	67.10 ± 0.57^a^	58.83 ± 0.88^b^	63.90 ± 0.91^ab^	66.19 ± 2.18^a^	67.03 ± 0.71^a^	69.79 ± 1.77^a^
WHC (g/g)	1.86 ± 0.01^a^	1.20 ± 0.09^b^	1.21 ± 0.08^b^	1.05 ± 0.06^b^	0.81 ± 0.06^c^	0.76 ± 0.04^c^
OHC (g/g)	2.11 ± 0.00^ab^	1.67 ± 0.03^c^	1.82 ± 0.12^bc^	1.67 ± 0.10^c^	1.89 ± 0.13^bc^	2.25 ± 0.17^a^
EAI (m^2^/g)	12.62 ± 0.19^a^	10.28 ± 0.49^c^	11.98 ± 0.50^ab^	12.40 ± 0.61^ab^	12.51 ± 0.04^ab^	11.04 ± 0.13^bc^
EC (%)	62.99 ± 3.68^ab^	55.00 ± 0.00^b^	61.53 ± 4.71^ab^	70.62 ± 0.81^a^	55.84 ± 0.28^b^	65.29 ± 3.87^ab^
ES (%)	96.32 ± 1.72^a^	100 ± 0.00^a^	98.18 ± 2.57^a^	77.21 ± 3.87^b^	97.27 ± 2.73^a^	99.56 ± 0.63^a^
FC (%)	79.02 ± 0.98^bc^	64.79 ± 3.36^c^	67.07 ± 1.22^c^	82.28 ± 1.80^bc^	90.65 ± 0.74^b^	108.52 ± 7.72^a^
FS (%)	83.75 ± 1.88^c^	79.86 ± 2.40^bc^	88.31 ± 1.88^ab^	89.41 ± 1.10^ab^	91.87 ± 0.28^a^	90.66 ± 0.69^a^

*Note*: Different superscript letters in the same line mean statistically significant differences between samples (*p* < 0.05).

Abbreviations: EAI, emulsifying activity index; EC, emulsion capacity; ES, emulsion stability; FC, foaming capacity; FS, foaming stability; OHC, oil holding capacity; WHC, water holding capacity.

High solubility, which means that a large proportion of the proteins can dissolve in water and remain available for interactions, is considered essential for various aspects of food processing, including protein functionality, sensory properties, and product stability (Yang et al. 2018). The solubility values observed in this study align with those previously reported for other legume proteins at neutral pH, which ranged from 57% to 65% (Hall and Moraru 2021; Choe et al. 2022). Although solubility in water was not improved by HHP technology, all concentrates presented values greater than 58%.

#### 3.2.2 WHC and OHC

The application of HHP on CBPC significantly decreased (*p* < 0.05) WHC (Table 2). As the pressure augmented (from 500 MPa), the decrease in WHC became more pronounced (*p* < 0.05), with WHC values falling below 1 g of water/g of protein. According to Meganaharshini et al. (2023), depending on pressure conditions and treatment duration, HHP can trigger a reduction in WHC. One possible explanation for this phenomenon could be the lack of polar amino acid groups on the protein surface after treatment at high pressures, as these groups are responsible for interactions between proteins and water (E. S. Tan et al. 2014; Mulla et al. 2022). At 600 MPa, significant structural changes were observed in CBPC, primarily on the protein surface, as indicated by higher *H*_0_ (Figure 5) and intrinsic fluorescence (Figure 6). These analyses revealed the exposure of more hydrophobic residues on the protein surface, which can be correlated to a decrease in solubility and WHC (Teixeira et al. 2023). Tan et al. (2014) also attributed the low WHC (1.65 g/g) of pinto bean protein to the high *H*_0,_ which limits the protein‐water interactions. As stated by Zhang et al. (2021), the rate of WHC of protein powders is influenced by particle size, number of polar groups, and surface hydrophilicity.

Regarding the OHC, almost all OHC contents were higher than those found for the WHC (Table 2). This would be expected because the carioca bean proteins are mainly globulins, molecules that are relatively hydrophobic (T. Lin and Fernández‐Fraguas 2020). Proteins rich in hydrophobic regions show a greater OHC (Zhang et al. 2021). In general, the sample treated at 600 MPa had the highest OHC value when compared to the other HHP‐treated CBPCs (Table 2). As mentioned previously, protein aggregates formed at lower pressures may have been disrupted at 600 MPa, allowing hydrophobic groups to be exposed on the protein surface and, consequently, enabling greater interaction between the oil and the protein of this treated concentrate (Carmo et al. 2023).

Pulse proteins exhibit wide variability in WHC and OHC across species and cultivars (Gravel and Doyen 2023). According to the literature, the WHC and OHC of legume protein range from 1.34 to 7.54 and 0.86 to 6.8 g/g, respectively (Rajpurohit and Li 2023; Gouvêa et al. 2023; E. S. Tan et al. 2014; K. K. Ma, Grossmann, et al. 2022). In the present work, all CBPC samples presented higher OHC than WHC, with OHC values falling within the reported legume range. The amount of oil retained by CBPC samples may contribute to the juiciness, mouthfeel, and carrying of fat‐soluble flavor compounds in food products (Day et al. 2022). This property can be particularly valuable in oil‐rich food formulations. Several food products, such as meat analogs, doughnuts, sausages, and bakery products, could benefit from the application of concentrates with high OHC (Gundogan and Karaca 2020; Meganaharshini et al. 2023). An illustrative example of the benefits of these characteristics can be seen in the recent introduction of vegetable chicken meat based on carioca bean protein in the Brazilian market. This innovative meat analog successfully replicates the texture and flavor profiles typically associated with traditional chicken meats (GFI Brasil 2023; GFI, The Good Food Institute 2020).

#### 3.2.3 Emulsifying Properties

The emulsifying properties of CBPC samples were assessed by their EAI, EC, and ES (Table 2). The EAI in the CBPC processed at 200 MPa significantly dropped when compared to most CBPC samples (*p* < 0.05), except for the sample treated at 600 MPa. EAI reflects the proteins’ ability to adsorb at the oil–water interface and contribute to emulsion formation (T. Lin and Fernández‐Fraguas 2020). HHP at 200 MPa led to protein aggregation (Figure 2), resulting in a significant reduction in solubility (Table 2). Solubility of proteins is essential for the emulsification because insoluble proteins lack the ability to adsorb and build a viscoelastic layer at the oil–water interface (M. Tan et al. 2023). Then, the aggregates present in the sample treated at 200 MPa did not contribute to emulsification due to hindered protein migration to the oil–water interface. In contrast, our previous study with DHP showed that pressures above 50 MPa increased the EAI of carioca bean proteins, an effect associated with increased solubility after processing (Santos et al. 2024). These findings highlight that pressure technology and its impact on protein solubility are key determinants of emulsifying performance.

The EC of the CBPC samples subjected to HHP did not show a significant difference from the control (*p* > 0.05). Besides that, the EC values fell within the range observed for various plant protein isolates (21%–88%) (K. K. Ma, Greis, et al. 2022). Regarding the ES, the sample treated at 400 MPa exhibited a statistically lower value compared to other CBPC samples, including the control. The ES for this sample was approximately 77%, whereas the stability of the other concentrates approached 100%, highlighting their significant potential for application in food products. The emulsifying performance of pulse proteins is closely related to surface charge, solubility, surface hydrophobicity, and the protein molecular flexibility (E. S. Tan et al. 2014). The reduction observed in the CBPC treated at 400 MPa can be attributed to the reduced molecular flexibility resulting from protein aggregation (Chen et al. 2019). The lowest ζ‐potential observed for this CBPC sample (Figure 3) possibly led to the formation of larger droplets and consequently the formation of a less stable emulsion (Gharibzahedi and Smith 2021).

#### 3.2.4 Foaming Properties

The application of physical treatments, such as HHP, has been shown to be effective in improving the foaming properties of pulse proteins (Amagliani et al. 2021). In the current study, the FC of CBPC was notably improved when the concentrate was subjected to HHP at 600 MPa, reaching a value of approximately 109% (Table 2). The enhancement can be primarily related to the smallest particle size and the greatest *H*_0_ found in this particular concentrate (Figures 2 and 6). An increase in *H*_0_, along with the disruption of insoluble protein aggregates, facilitates faster protein adsorption at the air–water interface, resulting in increased foam volumes (Fernando 2022; Neji et al. 2022). Other factors, such as protein solubility and ζ‐potential, also play a significant role in influencing the foaming properties (Dombrowski et al. 2017). This could potentially explain why the control sample did not exhibit a high FC, despite having similar *H*_0_ and particle size (*p* > 0.05) compared to the CBPC treated at 600 MPa.

Although FC in modified protein concentrates is generally increased, in some cases, the modifications can negatively impact FS compared to untreated samples (M. Tan et al. 2023). In a previous study with DHP, pressures up to 180 MPa enhanced the FC of carioca bean protein, but FS was compromised (Santos et al. 2024). Here, almost all CBPC samples treated by HHP had improved FS values, except that treated at 200 MPa (Table 2). The HHP treatment above 200 MPa likely generated a thicker and more cohesive film around the formed bubbles, leading to increased air bubble retention and a reduction in coalescence rate (Zhu et al. 2017; Kumar et al. 2022). Other bean varieties also exhibited improvements in FC and FS upon HHP treatment (Mulla et al. 2022).

In general, foam formation and stabilization by proteins primarily depend on their adsorption kinetics at the air–liquid interface, their ability to reduce surface tension, and their efficiency in covering and protecting air bubbles (Moll et al. 2022). A good FS associated with a high FC is ideal for products like creams, baked goods, and ice cream. In this context, the increased values of these parameters in CBPC samples indicate their suitability for applications in these types of food products (Gouvêa et al. 2023; Mulla et al. 2022).

### 3.3 Color Properties

Consumers heavily rely on color as a primary indicator for assessing the quality of a food product (K. K. Ma, Grossmann, et al. 2022). The luminosity (*L* ^*^) of CBPC samples experienced a noteworthy increase (*p* < 0.05) under pressure application up to 500 MPa. However, at 600 MPa, no significant difference was observed compared to the control (*p* > 0.05) (Table 3). HHP also had no noticeable impact on the green tinge (*a* ^*^) of the CBPC samples; conversely, yellowness (*b* ^*^) exhibited a significant increase at 400 MPa compared to other treated CBPC, but not in comparison to the control. Furthermore, chrome (*C* ^*^) and angle hue (*h*°) exhibited no significant differences among the concentrates (Table 3). All hue values fell within the red and yellow spectrum (60°–90°), signifying CBPC with a predominant inclination toward yellow coloring.

**TABLE 3:** Color parameters of carioca bean protein concentrate (CBPC) untreated and treated at 200, 300, 400, 500, and 600 MPa.

Parameters	Control	200 MPa	300 MPa	400 MPa	500 MPa	600 MPa
*L* ^*^	64.12 ± 1.13^c^	66.25 ± 0.81^ab^	67.69 ± 0.41^a^	67.52 ± 0.90^a^	66.93 ± 0.35^ab^	65.37 ± 0.62^bc^
*a* ^*^	3.19 ± 0.06^a^	3.08 ± 0.04^a^	3.08 ± 0.05^a^	3.14 ± 0.03^a^	3.12 ± 0.11^a^	3.14 ± 0.04^a^
*b* ^*^	6.59 ± 0.12^ab^	6.48 ± 0.13^b^	6.50 ± 0.04^b^	6.92 ± 0.07^a^	6.57 ± 0.12^b^	6.55 ± 0.11^b^
Chroma (*C* ^*^)	7.21 ± 0.21^a^	7.28 ± 0.22^a^	7.28 ± 0.16^a^	7.61 ± 0.05^a^	7.30 ± 0.16^a^	7.29 ± 0.09^a^
Hue (*h*°)	64.27 ± 0.72^a^	64.97 ± 0.68^a^	64.92 ± 0.24^a^	65.72 ± 0.66^a^	64.25 ± 0.38^a^	64.41 ± 0.63^a^

*Note*: Different superscript letters in the same line mean statistically significant differences between samples (*p* < 0.05).

**FIGURE 7:**
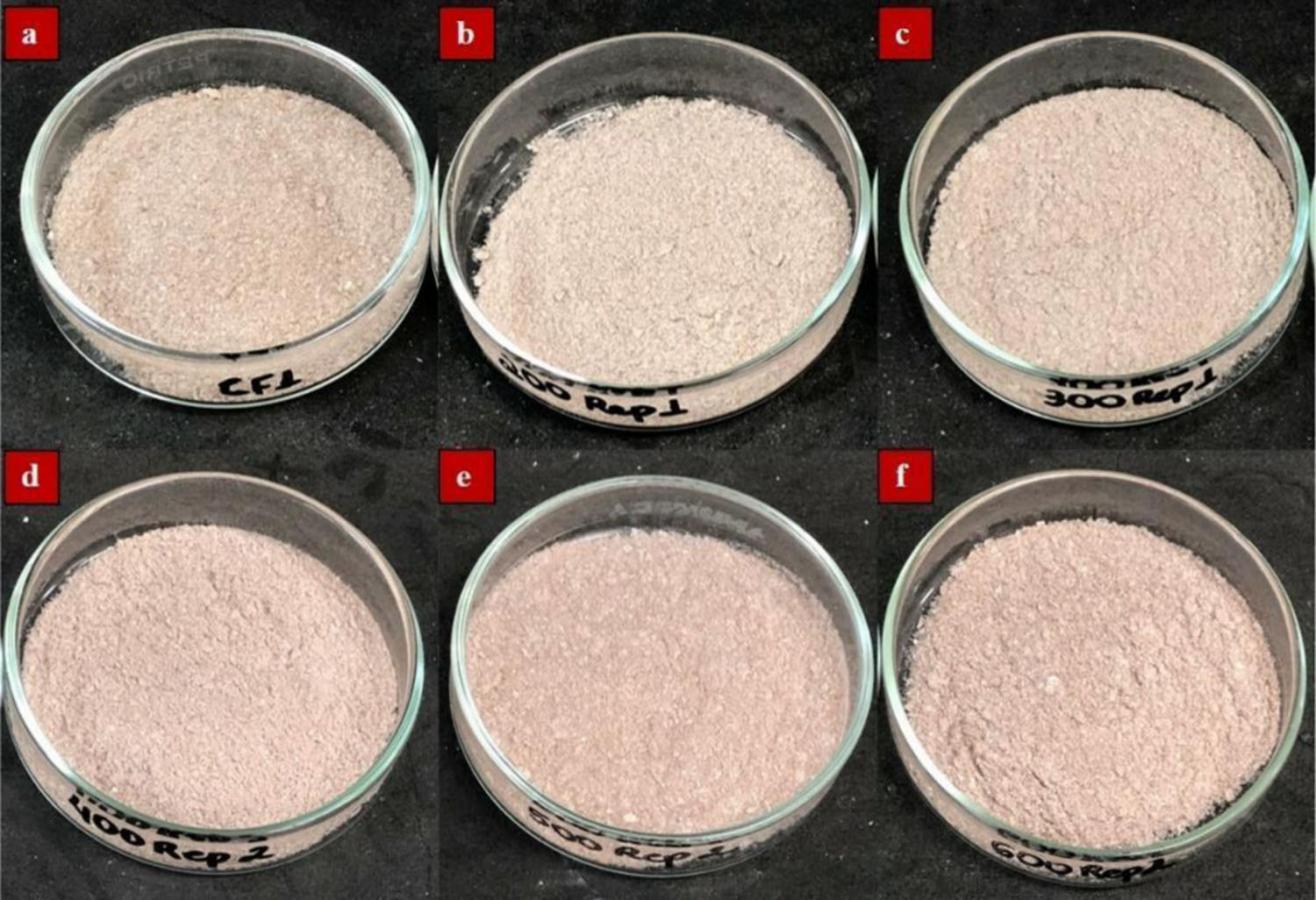
(a) Untreated and treated carioca bean protein concentrate (CBPC) subjected to HHP treatments at (b) 200, (c) 300, (d) 400, (e) 500, and (f) 600 MPa.

Addressing one of the major challenges in utilizing protein concentrates in plant‐based foods involves achieving a color that seamlessly integrates into the final product without negatively impacting consumer perception. In summary, CBPC samples showcased elevated *L* ^*^ and low *a* ^*^ and *b* ^*^ values, indicating protein powders with a whitish appearance and minimal pigment content (Figure 7). The ideal scenario for expanding the application of protein concentrates in various food products certainly includes these features (K. K. Ma, Grossmann, et al. 2022).

## 4 Conclusion

HHP treatment has proven to be an efficient tool for changing the properties of CBPC. Pressures from 200 MPa have demonstrated the capability to expose the hydrophobic groups within the protein, instigating the formation of aggregates, as evidenced by analyses of *H*_0_ and PSD. Conversely, under higher pressure (600 MPa), the pressure action mainly led to the breakdown of aggregates previously formed by the action of lower pressures. As a result, this breakdown produced a protein concentrate with structural characteristics relatively similar to the control; however, with significantly enhanced thermal stability and foaming properties. From a technological perspective, these enhancements are highly relevant for the development of aerated and emulsified plant‐based products.

Overall, HHP induces alterations in protein conformations, culminating in the development of unique characteristics in the CBPC while preserving its appealing color properties. As a result, this opens up new opportunities for the application of this protein ingredient in a diversity of products in the food industry, spanning from meat analogs and sausages to aerated or foamed foods, including whipped cream, aerated desserts, ice cream, and various bread goods.

Although the economic feasibility of HHP at an industrial scale remains challenging, this work provides a proof of concept demonstrating that HHP can be used as a clean‐label, non‐thermal tool to tailor bean protein functionality. Future studies should focus more deeply on its interfacial properties and nutritional characteristics to fully understand and exploit its value as a multifunctional ingredient in food applications.

## Author Contributions

**Fabiana Helen Santos**: conceptualization, data curation, formal analysis, investigation, methodology, visualization, validation, writing – original draft. **Ludmilla de Carvalho Oliveira**: methodology, validation, writing – review and editing, visualization, data curation, conceptualization, supervision. **Dirceu de Sousa Melo**: formal analysis. **Serafim Bakalis**: supervision, writing – review and editing, resources. **Marcelo Cristianini**: funding acquisition, resources, supervision, writing – review and editing.

## Conflicts of Interest

The authors declare no conflicts of interest.
